# In-Frame Variants in *STAG3* Gene Cause Premature Ovarian Insufficiency

**DOI:** 10.3389/fgene.2019.01016

**Published:** 2019-11-14

**Authors:** Wen-Juan Xiao, Wen-Bin He, Ya-Xin Zhang, Lan-Lan Meng, Guang-Xiu Lu, Ge Lin, Yue-Qiu Tan, Juan Du

**Affiliations:** ^1^Institute of Reproduction and Stem Cell Engineering, Central South University, Changsha, China; ^2^Genetic Center, Reproductive and Genetic Hospital of CITIC-Xiangya, Changsha, China; ^3^NHC Key Laboratory of Human Stem Cell and Reproductive Engineering, Central South University, Changsha, China

**Keywords:** premature ovarian insufficiency, *STAG3* gene, in-frame variant, fluorescence localization, co-immunoprecipitation

## Abstract

Premature ovarian insufficiency (POI) is a severe clinical syndrome defined by ovarian dysfunction in women less than 40 years old who generally manifest with infertility, menstrual disturbance, elevated gonadotrophins, and low estradiol levels. *STAG3* is considered a genetic aetiology of POI, which facilitates entry of REC8 into the nucleus of a cell and plays an essential role in gametogenesis. At present, only six truncated variants associated with POI have been reported; there have been no reports of an in-frame variant of *STAG3* causing POI. In this study, two novel homozygous in-frame variants (c.877_885del, p.293_295del; c.891_893dupTGA, p.297_298insAsp) in *STAG3* were identified in two sisters with POI from a five-generation consanguineous Han Chinese family. To evaluate the effects of these two variants, we performed fluorescence localization and co-immunoprecipitation analyses using *in vitro* cell model. The two variants were shown to be pathogenic, as neither STAG3 nor REC8 entered nuclei, and interactions between mutant STAG3 and REC8 or SMC1A were absent. To the best of our knowledge, this is the first report on in-frame variants of *STAG3* that cause POI. This finding extends the spectrum of variants in *STAG3* and sheds new light on the genetic origins of POI.

## Introduction

Premature ovarian insufficiency (POI), also known as premature ovarian failure, refers to hypergonadotropic hypogonadism in women younger than 40 years and is one of the major causes of female infertility, affecting at least 1–3% of adult women in the world (Bannatyne et al., 1990). Apart from menstrual disturbance (amenorrhea or oligomenorrhea for at least 4 months), the main symptoms of POI are reduced levels of estradiol and elevated plasma levels of follicle stimulating hormone (FSH) (>25 mIU/ml on two occasions, > 4 weeks apart) (Shelling, 2010; Gowri et al., 2015; Tucker et al., 2016; Webber et al., 2016). The etiology of POI is complex, such as that for autoimmune diseases, chemotherapy, and pelvic surgery, among which genetic defect was reported to be related to POI (Franca et al., 2019).

POI is a condition with extremely high genetic heterogeneity. A set of genes has been reported to be responsible for POI, including X-linked genes (e.g., *BMP15* and *FMR1*) and autosomal genes (e.g., *HFM1*, *FIGLA*, *FOXL2*, *STAG3*, and *FSHR*) (Rossetti et al., 2017). Stromal antigen 3 (*STAG3*) is a meiosis-specific gene that is restrictedly expressed in testes and ovaries in humans, and it plays an important role in gametogenesis and fertility (Houmard et al., 2009; Nogues et al., 2009; Garciacruz et al., 2010; Caburet et al., 2014). Variants of *STAG3* are rare, and only six truncated variants, three frameshift variants, two nonsense variants, and one splicing variant (Caburet et al., 2014; Stabej et al., 2016; Colombo et al., 2017; He et al., 2018a; Franca et al., 2019) (**Table 1**) associated with POI have been reported to date. However, it remains enigmatic whether in-frame variants of *STAG3* can cause POI and female infertility.

**Table 1:** The currently reported phenotypes and genotypes of *STAG3* gene in POI patients with 46,XX.

Patients	Genotype	Phenotype						References
		Menstrual history	FSH (mIU/ml)	LH (mIU/ml)	Estradiol (Pg/ml)	Ultrasonographic examination	Age at diagnosis (years)	
1	c.877_885del (p.293_295del) (Hom) c.891_893dupTGA (p.297_298insAsp) (Hom)	Primary amenorrhea	51	11.08	<10	Uterus and ovaries not visualized	18	This study
2	c.877_885del (p.293_295del) (Hom)c.891_893dupTGA (p.297_298insAsp) (Hom)	Primary amenorrhea	72.19	18.58	<10	Small uterus and small ovaries	21	This study
3	c.291dupC (p.Asn98Glnfs*2) (Het)c.1950C > A (p.Tyr650*) (Het)	Primary amenorrhea	89	37	<13	Infantile uterus; ovaries were not visualized	21	(Franca et al., 2019)
4	c.677C > G (p.Ser227*) (Hom)	Primary amenorrhea	48	23	19	NA	NA	(Colombo et al., 2017)
5	c.1573+5G > A (p.Leu490Thrfs*10) (Hom)	Primary amenorrhea	48.69	26	normal	Streak gonads and small uterus	19	(He et al., 2018a)
6	c.1573+5G > A (p.Leu490Thrfs*10) (Hom)	Primary amenorrhea	48.38	25.51	normal	NA	NA	
7	c.1947_48dupCT (p.Tyr650Serfs*22) (Hom)	Primary amenorrhea	136	31	<37	Normal uterus; bilateral streak gonads	NA	(Stabej et al., 2016)
8	c.1947_48dupCT (p.Tyr650Serfs*22) (Hom)	Primary amenorrhea	130	62	<37	Small uterus; streak gonads	NA	
9-12	c.968delC (p.Gln188Argfs*8) (Hom)	Primary amenorrhea	>45	>18	<22	Bilateral streak gonads	17–20 (4 sisters)	(Caburet et al., 2014)

POI, premature ovarian insufficiency; FSH, follicle stimulating hormone; LH, luteinizing hormone; Hom, homozygote variant; Het, heterozygote variant; NA, data not available.

In this study, we identified two novel homozygous in-frame variants of *STAG3* in a consanguineous Han Chinese family with POI by whole-exome sequencing (WES). Moreover, we performed *in silico* and *in vitro* functional analyses to reveal the relationship between the two variants and POI. To the best of our knowledge, this is the first report of in-frame variants of *STAG3* associated with POI, and the first example of performing *in vitro* functional analyses to functionally characterize in-frame variants of *STAG3*.

## Materials and Methods

### Patients and Blood Sampling

**Figure 1:**
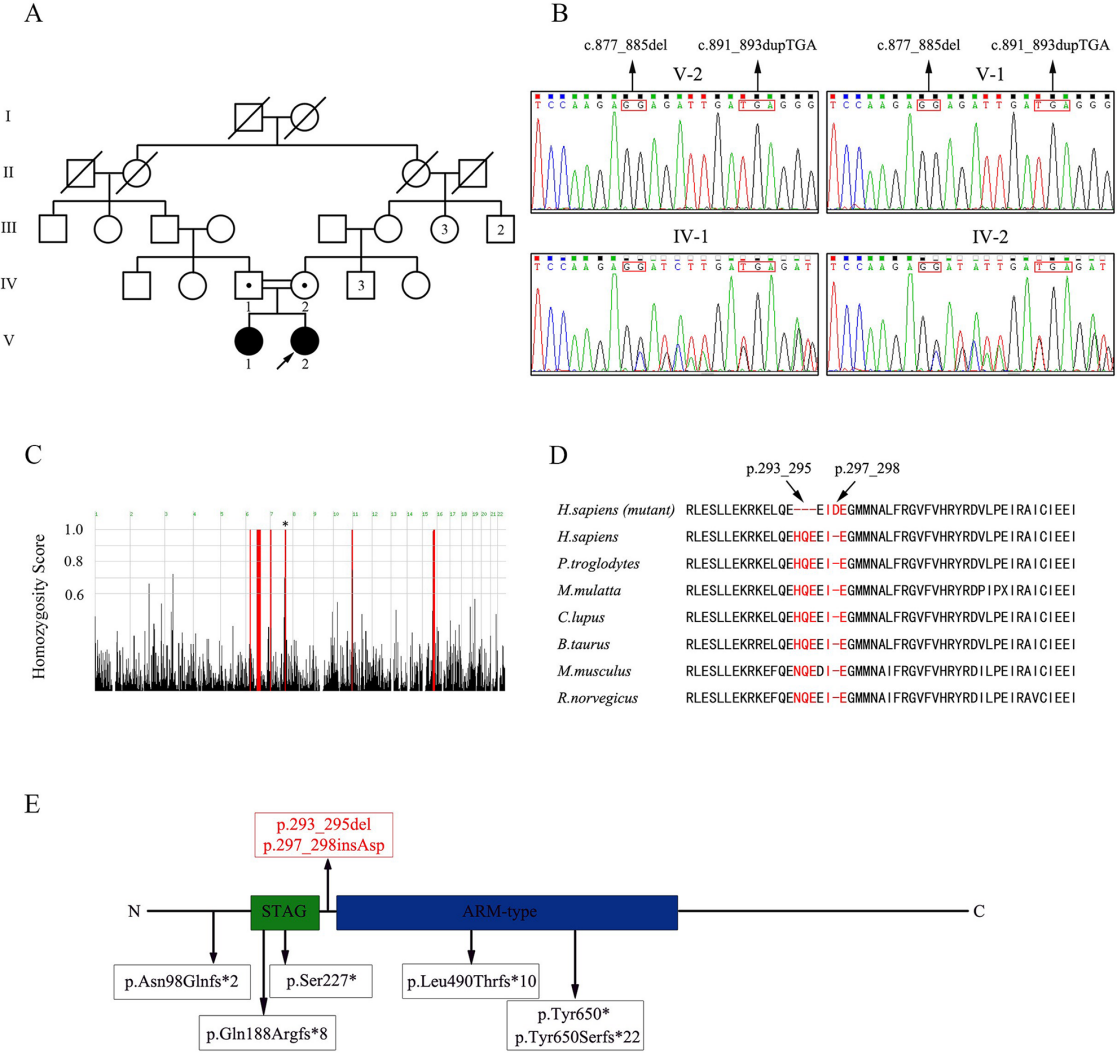
Pedigree of the consanguineous Han Chinese family with POI-associated *STAG3* variants. **(A)** Two second cousins (IV-1; IV-2) in generation four married each other and had two daughters (V-1; V-2) with POI. The number of siblings is written inside each symbol. The proband is marked with a black arrow. Filled symbols indicate other affected members. Open symbols indicate unaffected members. Heterozygous carriers are indicated with a dot in the middle of each symbol. Numbers are allotted to the family members whose DNA samples were used in this study. **(B)** Based on sequence chromatograms for *STAG3* (NM_001282716: c.877_885del; c.891_893dupTGA) in this family, the unaffected parents (IV-1; IV-2) are heterozygous carriers of two variants of *STAG3*, whereas the two affected sisters (V-1; V-2) are homozygous. **(C)** Homozygosity mapping of the proband. Homozygous regions with a remarkable signal are indicated in red. The asterisk indicates the location of *STAG3*. **(D)** Cross-species alignment reveals strong evolutionary conservation at positions 293_295 and 297_298 in *STAG3* (NP_036579.2). **(E)** The STAG3 protein consists of 1,225 amino acids, including a STAG domain and an ARM-type domain. The two variants discovered in this study are shown at the top of the figure, whereas previously reported variants are displayed at the bottom of the figure.

Four members of a five-generation consanguineous Han Chinese family with POI, namely, two affected sisters (V-1; V-2, **Figure 1A**) and two unaffected second-cousin parents (IV-1; IV-2, **Figure 1A**) participated in this study. Peripheral blood samples from the four individuals were collected, and genomic DNA (gDNA) was extracted using a QIAamp DNA Blood Midi Kit (Qiagen, Hilden, Germany). The four family members provided their written informed consent to participate in this study and agreed with the publication of this case report. This study was reviewed and approved by the ethics committee of the Reproductive & Genetic Hospital of CITIC-Xiangya of Central South University, China.

### WES, Variant Filtering, and Sanger Sequencing

The gDNA from the proband was subjected to WES following protocols described previously (He et al., 2018b). Whole exomes were captured using Agilent SureSelect version 4 (Agilent Technologies, Santa Clara, CA) and sequenced on a HighSeq2000 sequencing platform (Illumina, San Diego, California, USA). WES data analysis was performed using the Genome Analysis Toolkit (GATK) and consisted of removing adaptors, mapping WES raw reads to a reference human genome sequence (NCBI GRCh37, reference genome Hg19) using the Burrows–Wheeler Aligner (BWA), eliminating PCR duplicates, and sorting sequences using Picard (http://broadinstitute.github.io/picard/). Variant identification was performed using the GATK package according to the recommended best practices, including base recalibration variant calling with Haplotype Caller and variant quality score recalibration and annotation using ANNOVAR software.

All candidate variants needed to meet the following criteria: (i) absent or occurring at a frequency less than 0.01 in any of the following databases: 1,000 Genomes Variant Database, Exome Aggregation Consortium (ExAC), or NHLBI-GO Exome Sequencing Project (GO-ESP); (ii) homozygous variants were considered with priority; (iii) predicted to be deleterious; and (iv) relevant to the phenotype shared by the patients based on comprehensive expression data (expression in ovary), and model organism data (a female-lacking-oocytes phenotype presented in animal models).

Specific PCR primers targeting two variants of the *STAG3* gene (NM_001282716) were designed and validated. The sequences were as follows: 5′-AACCACATGCAGAGGGGTTAT-3′ and 5′-TCCAGCTGCATTAATTCTGGGA-3.′

### Plasmid Construction

The full-length coding sequence of *STAG3* was amplified from control human blood cDNA, and the *STAG3* product was cloned into pDsRed2-N1, resulting in STAG3 followed by DsRed2 protein, as STAG3-WT-DsRed2. Additionally, the cDNA encoding FLAG_3_-STAG3 was cloned into pcDNA3.1, namely, FLAG_3_-STAG3-WT. Variants c.877_885del and c.891_893dupTGA were separately or simultaneously introduced into the STAG3-WT-DsRed2 and FLAG_3_-STAG3-WT plasmid vectors using a Mut Express II Fast Mutagenesis Kit V2 (Vazyme, Guangzhou, China) to achieve site-directed mutagenesis. Altogether, six mutants were generated (Mut 1 indicates STAG3-p.293_295del-DsRed2 or FLAG_3_-STAG3-p.293_295del; Mut 2 indicates STAG3-p.297_298insAsp-DsRed2 or FLAG_3_-STAG3-p.297_​298insAsp; Mut 3 indicates STAG3-p.293_295del and p.297_298insAsp-DsRed2 or FLAG_3_-STAG3-p.293_295del and p.297_298insAsp). Moreover, cDNAs encoding REC8 and SMC1A were separately cloned into pEGFP-N1 and pcDNA3.1-HA. All expression constructs were sequenced to exclude unintended PCR-generated variants and confirm the presence of the desired variants.

### Cell Culture

Chinese hamster ovary (CHO) cells and human embryonic kidney (HEK) 293 cells were cultured in Dulbecco’s modified Eagle medium nutrient mixture F-12 (Ham) (DMEM/F12) (Gibco, Gaithersburg, MD, USA) with 10% fetal bovine serum (FBS) (Gibco, Gaithersburg, MD, USA) at 37°C in a humidified 5% CO_2_ incubator.

### Fluorescence Localization

According to the manufacturer’s instructions for Lipofectamine 3000 (Thermo Fisher Scientific, Carlsbad, CA, USA), CHO cells were transfected with expression vectors containing wild-type *STAG3-DsRed2* and *REC8-EGFP* plasmids or mutated *STAG3-DsRed2* and *REC8-EGFP* plasmids. CHO cells transfected with empty vector (EV; without *STAG3*) and *REC8-EGFP* plasmids were used as negative controls. CHO cells were fixed for fluorescence localization after culture for 48 h. 4’,6-Diamidino-2-phenylindole (DAPI) (Beyotime) was used to visualize DNA. All fluorescent images were captured on an orthostatic fluorescence microscope (Olympus, Japan).

### Co-Immunoprecipitation (Co-IP)

Co-IP was performed as described previously (Wolf et al., 2018). Briefly, HEK293 cells were transfected with expression vectors containing wild-type *FLAG* *_3_*-*STAG3* and *REC8-EGFP* plasmids (or SMC1A-HA plasmids) or mutated *FLAG* *_3_*-*STAG3* and *REC8-EGFP* plasmids (or SMC1A-HA plasmids), according to the manufacturer’s instructions for Lipofectamine 3000 use. Untransfected HEK293 cells were used as a negative control. After incubation for 36 h, the culture medium was supplemented with 0.2 µg/ml nocodazole (Sigma, Louis, MO, USA) for prometaphase arrest of the cells. Total protein was extracted using RIPA lysis buffer (Thermo Fisher Scientific) after 12 h, and then, western blotting was performed to detect the expression of STAG3, REC8, and SMC1A using anti-FLAG, anti-GFP, and anti-HA (Proteintech) antibodies, respectively, at a 1:1,000 dilution. Next, immunoprecipitation was performed using Dynabeads Protein G (Invitrogen) following the manufacturer’s instructions.

## Results

### Clinical Features of the Affected Individuals

The proband (V-2, **Figure 1A**) initially went to the clinic because of primary amenorrhea and was diagnosed with POI at 18 years old. Her breast development was at Tanner stage I, although she had a normal height (160 cm) and weight (50 kg). The results of hormonal studies showed that her serum FSH level was high (51 mIU/ml, normal range 2–22 mIU/ml), whereas her serum estradiol level was low (< 10 pg/ml, normal range 30–190 pg/ml), and her anti-Müllerian hormone (AMH) level was too low to be detected. She had a normal level of luteinizing hormone (LH; 11.08 mIU/ml, normal range 1–19 mIU/ml). Her uterus and ovaries were not visualized upon ultrasonographic examination, and she had an abnormal vulva without labia minora. The proband did not accept hormonal replacement therapy (HRT) because of the increased prevalence of invasive breast cancer after HRT (Biscup, 2003).

The proband’s affected elder sister (V-1, **Figure 1A**) showed normal growth and development but presented with POI when she was 21 years old. Similar to the proband, she went to the hospital because of primary amenorrhea. Her serum FSH level was elevated (72.19 mIU/ml, normal range 2–22 mIU/ml), whereas her serum estradiol level was low (< 10 pg/ml, normal range 30–190 pg/ml), and her LH level was normal (18.58 mIU/ml, normal range 1–19 mIU/ml). Ultrasonographic examination showed that her uterus was anteflexed and small (23×10×20 mm). Both ovaries were also small: the left ovary was 14×10×11 mm, and the right one was 15×11×13 mm, consistent with the size of streak gonads. After receiving HRT with progesterone for 3 months, she showed normal menses, and her uterus grew (36×20×35 mm) to a size close to that of a normal adult-sized uterus.

Additionally, both sisters had a normal karyotype (46, XX) and FMR1 CGG repeats, and no associated adrenal or thyroid autoimmune disorders were found.

### Identification of Two Homozygous In-Frame Variants of *STAG3*

WES of the proband (V-2, **Figure 1A**) resulted in approximately 139,631,574 raw reads with a mean 182.73-fold depth in the target regions, revealing high-quality sequencing (**Supplementary Table 1**). For rare inherited diseases, the frequency of possible pathogenic variants should be very low in a healthy population. Therefore, the results were screened against minor allele frequency (MAF) > 1% in public SNP and indel databases for variants predicted to be deleterious or to result in loss of function. Homozygous variants were preferentially considered candidates because the patients were from a consanguineous family. Finally, we focused on the phenotypic relevance for POI in the proband. Only two homozygous variants of *STAG3* (NM_001282716: c.877_885del, p.293_295del; c.891_893dupTGA, p.297_298insAsp) fulfilled these criteria (**Supplementary Table 2**).

The two homozygous in-frame *STAG3* variants were confirmed by Sanger sequencing and were also detected in her affected sister (**Figure 1B**). Their unaffected parents were heterozygous carriers of the two variants (**Figure 1B**). In addition, homozygosity mapping indicated that the two *STAG3* variants were not located in heterozygous regions (**Figure 1C**) and that positions p.293_295 and p.297_298 are highly conserved across different species (**Figure 1D**).

According to the American College of Medical Genetics and Genomics (ACMG) standards and guidelines for the interpretation of variations (Richards et al., 2015), the two in-frame variants of STAG3 (c.877_885del, p.293_295del; c.891_893dupTGA, p.297_298insAsp) are both classified as likely pathogenic variants. Therefore, we speculated that both homozygous *STAG3* variants were candidates as causes of POI.

### Effects of Mutant STAG3 Proteins on REC8 Localization in CHO Cells

**Figure 2:**
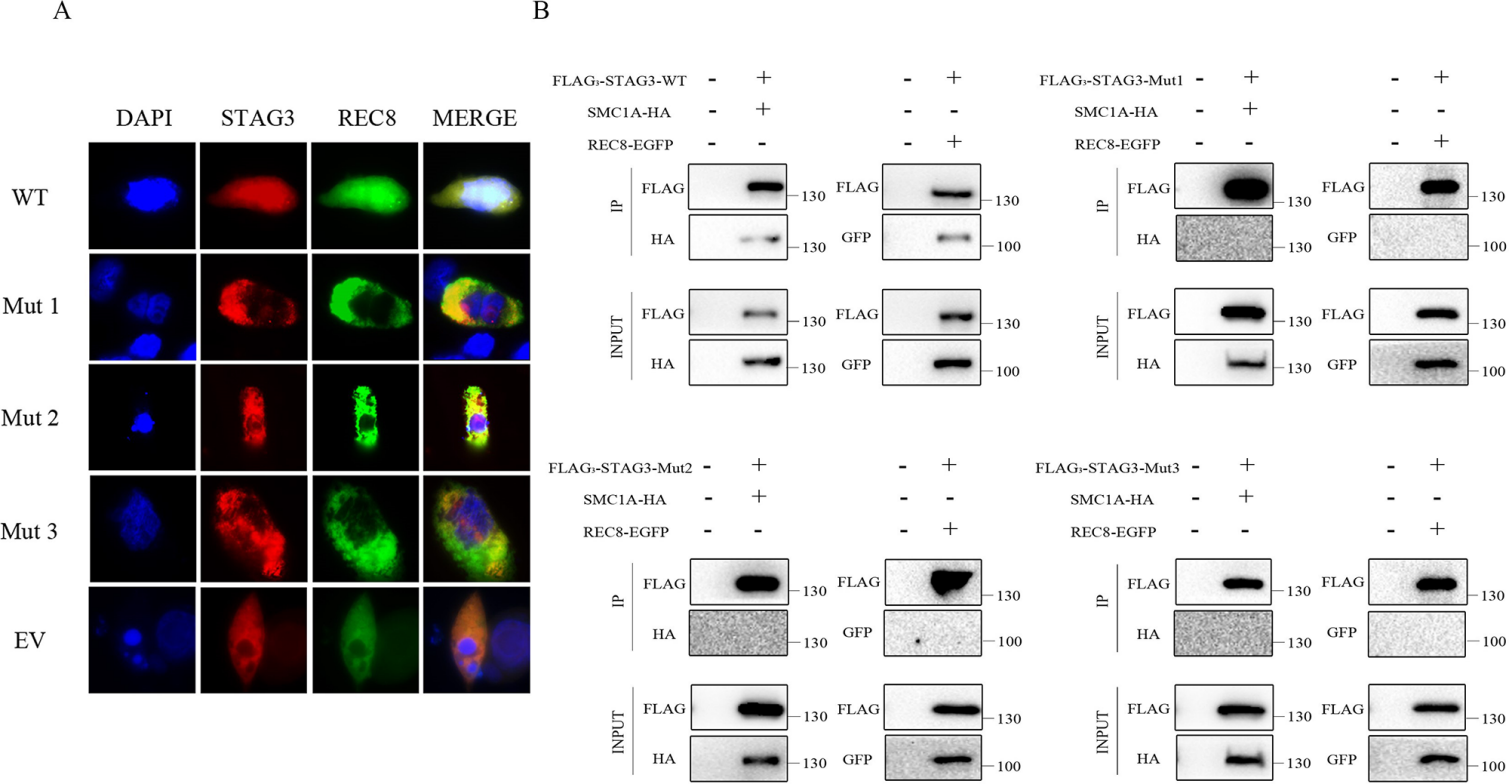
Fluorescence localization and Co-IP analyses of the two in-frame variants of *STAG3*. **(A)** CHO cells were transiently transfected with a plasmid encoding STAG3-DsRed2 and REC8-EGFP, mutant STAG3-DsRed2 and REC8-EGFP, or an empty vector and REC8-EGFP. After culture for 48 h, cells were fixed for fluorescence localization. Blue fluorescence indicates DAPI staining in nuclei. The three mutant STAG3 proteins were restricted to the cytoplasm and could not assist REC8 in entering nuclei, resulting in aberrant localization of REC8. **(B)** HEK293 cells were transiently transfected with the plasmid encoding FLAG_3_-STAG3 (or mutant FLAG_3_-STAG3) and REC8-EGFP, or FLAG_3_-STAG3 (or mutant FLAG_3_-STAG3) and SMC1A-HA. Untransfected HEK293 cells were used as the negative control. The culture medium was supplemented with nocodazole for prometaphase arrest of the cells after 36 h. Total protein was extracted after 12 h for Co-IP analysis. Wild-type STAG3 interacted with both REC8 and SMC1A, whereas there was no interaction between the three mutant STAG3 proteins with REC8 or SMC1A. WT indicates the plasmid encoding the wild type STAG3, Mut 1 indicates the plasmid encoding c.877_885del (p.293_295del) STAG3, Mut 2 indicates the plasmid encoding c.891_893dupTGA (p.297_298insAsp) STAG3, Mut 3 indicates the plasmid simultaneously encoding c.877_885del (p.293_295del) and c.891_893dupTGA (p.297_298insAsp) STAG3, and EV indicates the empty vector (without *STAG3*).

Co-expression of wild-type STAG3 and REC8 is necessary for REC8 to enter nuclei. A fluorescence localization analysis was performed to evaluate the effects of the two variants. As expected, REC8 localized to nuclei, as did the will-type STAG3 protein, when CHO cells were transfected with expression vectors containing wild-type *STAG3* and *REC8* plasmids; (**Figure 2A**). In contrast, REC8 localized exclusively in the cytoplasm (**Figure 2A**) when CHO cells were co-transfected with the *REC8* construct and empty vector or mutant *STAG3* construct. In the latter scenario, nearly all of the mutant STAG3 proteins were excluded from nuclei (**Figure 2A**).

### Lack of Interactions Between Mutant STAG3 and REC8 or SMC1A

We then performed a Co-IP analysis to determine the pathogenicity of the two variants. As shown, wild-type STAG3 interacted with both REC8 and SMC1A as previously reported (Prieto et al., 2001; Wolf et al., 2018) (**Figure 2B**). Additionally, when we directly examined the ability of the three transiently expressed mutant STAG3 proteins (p.293_295del; p.297_298insAsp; p.293_295del, and p.297_298insAsp) to associate with REC8 or SMC1A, the results revealed that there was no interaction between the three mutant STAG3 proteins and REC8 or SMC1A (**Figure 2B**).

## Discussion

In the present study, two novel homozygous in-frame variants (c.877_885del, p.293_295del; c.891_893dupTGA, p.297_298insAsp) of *STAG3* were identified in a consanguineous Han Chinese family with POI. A fluorescence localization analysis was employed to evaluate the effects of the two variants and revealed that the mutant STAG3 proteins led to aberrant localization of REC8. Furthermore, interactions between the mutant STAG3 proteins and REC8 or SMC1A were absent, which was confirmed by Co-IP analysis. Therefore, both in-frame variants were shown to be deleterious and associated with POI in this family.

The STAG3 protein contains two domains: the STAG domain and the armadillo (ARM)-type domain. The ARM-type domain is located after the STAG domain, which is predicted to interact with nucleic acid or another protein (Caburet et al., 2014) (**Figure 1E**). At present, six *STAG3* variants have been reported in five families with POI (Caburet et al., 2014; Stabej et al., 2016; Colombo et al., 2017; He et al., 2018a; Franca et al., 2019) (**Table 1**). All six variants are truncated variants that severely disrupt the STAG domain and/or ARM-type domain. Even c.1573+5G > A is a splice site variant, with RT-PCR revealing that the resulting mutant STAG3 protein is truncated (p.Leu490Thrfs*10) (He et al., 2018a), leading to destruction of the ARM-type domain. In our study, both variants (p.293_295del and p.297_298insAsp) are in-frame variants, and neither localizes to the STAG or ARM-type domain; both variants are positioned between these two domains (**Figure 1E**). Therefore, the STAG and ARM-type domains are not disrupted, in theory. However, the p.293_295 and p.297_298 sites are highly conserved across species (**Figure 1D**), and non-truncating variants located between two domains have also been reported to impair protein function and lead to disease (Xia et al., 2015). Therefore, we postulate that the two variants are pathogenic, and that the region connecting the STAG and ARM-type domains is significant for STAG3 function.

To verify the pathogenicity of the two variants, we performed *in vitro* functional analyses to determine whether STAG3 protein function was impaired. *STAG3* encodes a meiosis-specific subunit of cohesin, a multiprotein complex that plays an essential role in the proper pairing of chromosomes, sister chromatid cohesion, and chromosome segregation (Caburet et al., 2014; Wolf et al., 2018). Furthermore, STAG3 has been reported to interact with the cohesion subunits REC8 and SMC1A, and the entry of REC8 into nuclei requires STAG3 (Prieto et al., 2001; Wolf et al., 2018). The results of the fluorescence localization analysis in this study showed that none of the three mutant STAG3 proteins could assist REC8 in entering nuclei and that nearly all of the STAG3 mutants were restricted to the cytoplasm. In addition, there were no interactions between the mutant STAG3 proteins and REC8 or SMC1A observed in the Co-IP analysis. Therefore, we propose that the two in-frame variants of *STAG3* lead to STAG3 dysfunction and are responsible for POI in the two study patients.

The proband’s affected sister is sterile and has a small uterus and streak gonads, which is consistent with patients with POI caused by other *STAG3* variants (**Table 1**). In contrast, the uterus and ovaries of the proband in our study were not visualized by ultrasonography (**Table 1**). The difference in phenotype between the proband and her affected sister might be explained by the proband’s affected sister receiving HRT before coming to our hospital. Her uterus became larger after 3 months of HRT, which is consistent with patients with POI caused by variants in *MCM8* and *BRCA2* (Tenenbaumrakover et al., 2015; Weinbergshukron et al., 2018). Both *MCM8* and *BRCA2* have been shown to be associated with meiosis and female infertility (Moynahan et al., 2001; Rodriguezmari et al., 2011; Gambus and Blow, 2013), and *STAG3* is a meiosis-specific gene involved in female infertility. Furthermore, *MCM8* and *BRCA2* have been associated with ovarian dysgenesis in humans (Moynahan et al., 2001; Rodriguezmari et al., 2011; Gambus and Blow, 2013). Female *stag3* ^−/−^ mice also present with severe and very early ovarian dysgenesis (Caburet et al., 2014). Therefore, we suggest that *STAG3* might also be related to ovarian dysgenesis in humans.

In summary, to the best of our knowledge, we are the first researchers to report in-frame variants of *STAG3* that cause POI and to verify the role of in-frame *STAG3* variants in POI pathogenicity through *in vitro* functional analyses of cell model. Our findings extend the mutational and phenotypic spectrums of *STAG3* and have important implications for genetic counseling of patients with POI. However, to confirm the relationship between *STAG3* and ovarian dysgenesis in humans, further study with a large sample size is needed.

## Data Availability Statement

The raw data supporting the conclusions of this manuscript will be made available by the authors, without undue reservation, to any qualified researcher.

## Author Contributions

JD and Y-QT designed the study. W-JX, W-BH, Y-XZ and L-LM performed the variant analysis of *STAG3*. W-JX and W-BH carried out the evaluation of the pathogenicity of variations by *in vitro* cell model functional analyses. G-XL and GL worked on the clinical study. W-JX, W-BH, Y-QT and JD wrote the paper. All authors read and approved the final manuscript.

## Funding

This work was supported by grants from the National Key Research & Developmental Program of China (2018YFC1004900), the National Natural Science Foundation of China (81771645 and 81971447), the Hunan Provincial Natural Science Foundation of China (2019JJ51006), the Scientific Research Foundation of the Health Committee of Hunan Province (C2019193), the science and technology major project of the ministry of science and technology of Hunan Province, China (2017SK1030), and the Scientific Research Foundation of Reproductive and Genetic Hospital of CITIC-Xiangya (YNXM-201915, YNXM-201913, YNXM-201912, YNXM-201916).

## Conflict of Interest

The authors declare that the research was conducted in the absence of any commercial or financial relationships that could be construed as a potential conflict of interest.
