# Supplementary material for: In-Frame Variants in STAG3 Gene Cause Premature Ovarian Insufficiency
Source: Front Genet. 2019 Nov 14;10:1016. doi: 10.3389/fgene.2019.01016 (PMC6868891; doi:10.3389/fgene.2019.01016)
Supplement: Supplementary file 1 [file Table_1.docx]

**Supplementary table 1. Summary of whole exome sequencing data from the proband in this family.**

| Sample ID | V-2 (proband) |
| --- | --- |
| Initial bases on target | 50,390,601 |
| Raw reads | 139,631,574 |
| Clean reads | 138,016,040 |
| Total effective bases (Mb) | 17,198.90 |
| Effective sequences on target (Mb) | 9,207.73 |
| Capture specificity (%) | 53.54 |
| Mapping rate on genome (%) | 99.98 |
| Coverage of target region (%) | 99.69 |
| Mean depth of target region (X) | 182.3 |
| Fraction of target region covered >=1X (%) | 99.86 |
| Fraction of target region covered >=4X (%) | 99.80 |
| Fraction of target region covered >=10X (%) | 99.66 |
| Fraction of target region covered >=20X (%) | 99.20 |
| Clean data reads (%) | 97.57 |
| Clean read1 Q20 (%) | 98.57 |
| Clean read2 Q20 (%) | 96.63 |
| Clean read1 Q30 (%) | 95.65 |
| Clean read2 Q30 (%) | 91.85 |
| GC (%) | 48.98 |
| Gender test result | Female |
