# Supplementary material for: In-Frame Variants in STAG3 Gene Cause Premature Ovarian Insufficiency
Source: Front Genet. 2019 Nov 14;10:1016. doi: 10.3389/fgene.2019.01016 (PMC6868891; doi:10.3389/fgene.2019.01016)
Supplement: Supplementary file 2 [file Table_2.docx]

**Supplementary table 2. Candidate pathogenic gene presents in the proband in this family.**

| Gene | RefSeq ID | Chr | Position^1^ | dbsnp 142 | Function | Amino Acid Alteration | 1000G | GO-ESP | ExAC |
| --- | --- | --- | --- | --- | --- | --- | --- | --- | --- |
| *STAG3* | NM_001282716 | Chr7 | 99792935 | NA | In-frame | c.877_885del  (p.293_295del) | NA | NA | NA |
| *STAG3* | NM_001282716 | Chr7 | 99792954 | NA | In-frame | c.891_893dup  (p.297_298insAsp) | NA | NA | NA |

|  | CADD | MGI | Phenolyzer |
| --- | --- | --- | --- |
| c.877_885del  (p.293_295del) | 22.3 | Reproductive system phenotype;  endocrine/exocrine gland phenotype;  cellular phenotype;  behavior/neurological phenotype;  growth/size/body phenotype;  hematopoietic system phenotype;  skeleton phenotype | Premature ovarian insufficiency |
| c.891_893dup  (p.297_298insAsp) | 21.4 |  |  |

Note: CADD score: amino acid substitution is predicted damaging if the score is >20. MGI: Mouse Genome Informatics. NA: not available.
